# The Illness Narratives of Children and Young People With Spinal Muscular Atrophy: A Scoping Review

**DOI:** 10.1111/jan.70468

**Published:** 2025-12-24

**Authors:** Marcela González‐Agüero, Constanza Quezada, Valentina Turén, Josefa Camelio, Martina De Filippi, Caroline Bradbury‐Jones, Julie Taylor

**Affiliations:** ^1^ School of Nursing Pontificia Universidad Católica de Chile Santiago de Chile Chile; ^2^ Instituto Oncológico Fundación Arturo López Pérez Santiago de Chile Chile; ^3^ School of Anthropology Pontificia Universidad Católica de Chile Santiago de Chile Chile; ^4^ Department of Nursing and Midwifery University of Birmingham Birmingham UK

**Keywords:** adolescent health, child health services, lived experience, narrative analysis, PAGER framework, right to health, SMA

## Abstract

**Aim(s):**

This review seeks to explore the illness narratives of children and young people focusing on their healthcare trajectories; the right to health; and the kind of stories told about them.

**Design:**

This scoping review adopts a narrative approach to analyse how the illness experience of Spinal Muscular Atrophy is represented in the literature, moving beyond biomedical descriptions to consider sociocultural and historical dimensions. We explore how global and local forces shape everyday life and therapeutic possibilities for people with this condition.

**Methods:**

Four online databases were used to identify papers published between 2014 and 2024 in English and Spanish. The analysis process was guided by the PAGER Framework.

**Results:**

Twenty‐one articles met the criteria for the review, mainly published in the Global North. Following organisation of Patterns, findings were categorised into three themes: (1) Parents as storytellers of young people's life trajectories; (2) Tropes about everyday life with Spinal Muscular Atrophy; and (3) The right to health as a narrative terrain. Findings show that access to medical treatment, information, and healthcare coverage poses difficulties when navigating the healthcare system with little institutional support.

**Conclusions:**

The voices of individuals with Spinal Muscular Atrophy are rarely reported, often represented by their parents. There are opportunities to develop strategies that enhance the experiences of children and young people when seeking care, which should have a rights‐based, intersectional, and family‐centred approach.

**Impact:**

This review highlights the need to listen to children and young people's voices, offer support to caregivers, and further explore the right to health in the Global South.

**Patient or Public Contribution:**

The interpretation of the findings was enriched by the involvement of patients, who participated as advisors for the research team. Their contributions ensured the research remained aligned with concerns and priorities informed by lived experience of the disease.

## Summary

- What already is known: While multiple studies have focused on the progression and treatment of Spinal Muscular Atrophy, little has been said about the illness narratives of those affected by it.
- What this paper adds: Most literature has been produced in the Global North, leaving the experience of those from the South invisible. Parents tend to be the main narrators of children and young people's experiences, focusing on emerging clinical and socioeconomic challenges while trying to navigate their children's right to health.
- Implications for practice/policy: As mothers emerge as the main caregivers, it is essential to design services to accompany them, especially when they lack other support networks. It is also recommended that when working with young people, healthcare professionals should try to establish a relationship with them without intermediaries if possible, so that they can express their concerns and wishes on their own terms.

## 1 Background

Spinal Muscular Atrophy (SMA) is a genetic rare disease with autosomal recessive inheritance, characterised by progressive proximal muscle weakness and skeletal muscle atrophy (Nishio et al. 2023). It is caused by a genetic defect that reduces the production of the survival motor neuron (SMN) protein, which is essential for the neurons' maintenance and functionality. As a result, people with SMA experience progressive muscle degeneration, significantly impacting their ability to walk, speak, swallow, and breathe (Cleveland Clinic 2022). As the disease advances, the muscles responsible for these essential functions progressively weaken and atrophy, leading to severe disability or premature death (Cleveland Clinic 2022). Worldwide, SMA is recognised as the leading cause of hereditary infant mortality (Nishio et al. 2023). Thus, it has significant global relevance due to its impact on families, children, and the health systems (Chan et al. 2023). Its prognosis is closely connected to the type of SMA (Chung et al. 2004), being very poor for those affected by type I SMA (without treatment their life expectancy is less than six months) and relatively good for those affected by type IV (with a 100% survival rate at age 40).

Since the first documented case of SMA in 1891, research and medical advancements have progressed through three major phases (Nishio et al. 2023). The first phase (1891–1994) focused on identifying and classifying the disease, ultimately leading to the recognition of five subtypes: Types 0–IV. The second phase (1995–2015) began with the cloning of the SMN genes and concentrated on the development of drug therapies. The third and current phase (2016–present) started with the introduction of FDA‐approved treatments (essential for the survival of children affected by type I SMA, the most severe and common type of SMA) and includes the implementation of newborn screening programs for early diagnosis.

Rare diseases present significant challenges to health systems, including limited medical knowledge, high treatment costs, a lack of clinical expertise, and uncertainty about the cost‐effectiveness of available therapies (Valdez et al. 2017). In response, patient advocacy groups have played a pivotal role in positioning access to treatment as a fundamental health right and demanding state intervention to ensure equitable access to life‐saving therapies (Encina et al. 2019). This struggle reflects broader debates about the right to health and the responsibility of governments to guarantee universal access to medical care.

### 1.1 Rare Diseases and the Right to Health

Rare diseases call for a human rights‐based approach to their study, as people affected by these conditions are permanently trying to claim their rights when accessing healthcare services. According to international human rights instruments, every person, independent of sex, religion, age, ethnicity, or nationality, is entitled to health services, medicines, and equipment that are available, accessible, acceptable, and of good quality (United Nations General Assembly 1948). The right to health entails a powerful legal and public health tool subjected to the principle of progressive realisation, meaning that states must take actions to fulfil it according to their level of development and financial resources, monitoring progress and avoiding retrogressive measures (United Nations Committee on Economic, Social and Cultural Rights 1990).

To clarify what the right entails in practice, in 2000, the United Nations' Committee on Economic, Social and Cultural Rights included an overarching framework on “Availability, Accessibility, Acceptability and Quality” of health services and medicines (Montel et al. 2022). Following this regulation, in 2009, the World Health Organization and the Office of the High Commissioner for Human Rights published guidance on the implementation of a human rights‐based approach to health systems. Currently, around 140 countries worldwide recognise the right, with access to medicines being a key aspect.

This is particularly relevant to those with SMA, as the emergence of pharmaceutical options has opened new possibilities for their future. Furthermore, it has brought the right to health, and more specifically, the right to pharmaceuticals to the fore. The first FDA‐approved treatment was Spinraza in 2016 in the U.S., followed by its approval in Europe and Japan in 2017. This drug enhances SMN protein production and improves motor neuron function, thereby enhancing patients' quality of life, having an annual cost for the first 12 months of USD $7,000,000 approximately. In 2019, Zolgensma, a gene therapy designed to replace the defective SMN1 gene, was approved in the U.S. and later in Europe and Japan in 2020. This therapy improves muscle strength and survival rates in children with Type I SMA and is currently the most expensive single‐dose drug on the market, with a price of USD $2.1 million per person. The most recent approval was Evrysdi, authorised in the U.S. in 2020 and in Europe and Japan in 2021. This oral therapy increases SMN protein levels, helping to mitigate disease symptoms, with an annual cost of USD $350,000 approximately. While Spinraza and Evrysdi are considered palliative treatments that improve quality of life, they impose a significant financial burden (Canadian Agency for Drugs and Technologies in Health 2018).

In this context of pharmaceutical advances and innovation, the drugs prices operate under the laws of supply and demand in the pharmaceutical market, affecting access for people with SMA. The elevated prices make drugs unattainable for most in the Global South, generating profound inequalities within social groups and across countries (Buckle et al. 2024). When medicines on the market are simply too expensive, people are pushed to take legal action to claim their right (Biehl 2016; Socal et al. 2020), securing access to the treatment they need at the individual level but losing a collective perspective on the understanding of the right to health (Khachigian 2020).

### 1.2 A Narrative Approach to Explore the Experiences of People Living With SMA

In this scoping review we use a narrative to analyse the included literature. The illness experience goes beyond understanding a disease as a set of symptoms, signs and physio‐pathological processes, considering the sociocultural and historical context, as well as the ideas, values and practices connected to ill‐health. We believe that the case of SMA offers an opportunity to analyse the connections between the global and local forces upon everyday life, as the recent development of high‐cost drugs has profoundly changed the therapeutic landscape for this group, their future possibilities and imagined projects. In doing so, we bring to the fore the stories (or narratives) of people with SMA, paying special attention to what is said about them and their families, how this is said, when and by whom.

Healthcare contexts are particularly suited for using narrative analysis as patients, their relatives and clinicians regularly share ill‐health related stories with each other. These accounts involve a complex fabric of beliefs, values, emotions, identities, knowledge, attitudes and behaviours deeply rooted in a historical and geographical context (Vindrola‐Padros and Johnson 2014). As Lawlor and Mattingly (2000) have explained, “stories concern action, more specifically human action, and particularly social interaction. Stories have plots […] while they unfold in time […] they reveal a ‘sense of the whole’” (6). Attending to narratives can help clinicians engage with their patients and enrich the diagnosis and treatment process (Rushforth et al. 2021). Moreover, Charon has argued that clinicians are required to develop a narrative competence to “acknowledge, absorb, interpret and act on the stories and plights of others” (Charon 2001, 1897).

In this review, we followed Frank's typology (Frank 1995, 2013), which proposes three kinds of illness narratives: the restitution, the chaos, and the quest narrative. The first one tends to be the most common and culturally desirable narrative, as it focuses on a full recovery after an illness experience; the person returns to normal life and finds a cure. The second narrative differs from the restitution one as it involves permanent suffering and no apparent resolution. The individual's experiences are marked by a sense of vulnerability, uncertainty about the future, and a lack of control over the process. Finally, the third narrative type suggests a different stage in the patient's trajectory, describing a process of acceptance of the illness despite the absence of a cure. The individual recounts a transformative journey where they face suffering, assign new meaning to the experience, and in some cases, construct a new identity.

While a great deal has been written about children and young people with SMA from a biomedical perspective, the literature about their own experiences is more limited (Kirk and Hinton 2019), and according to our knowledge, there have been no reviews conducted of children and young people with SMA from a narrative perspective. This scoping review took place during the first year of implementation of a larger three‐year ethnographic study that the first author is conducting in Chile (*Fondecyt Iniciación* ID11240547). The main study will answer the following questions: How do children and young people with SMA and their families understand and co‐create citizenship and the right to health? What kind of narratives and life trajectories emerge from this process? Thus, the scoping review seeks to map relevant literature on the topic to situate the study in the global context.

## 2 Methodology

This review (Peters et al. 2021) summarises current literature about the life experiences and healthcare trajectories of children and young people with SMA. We selected this methodology with the aim of exploring the breadth and depth of the literature, identifying the approaches and foci of previous studies, as well as the gaps and advances. Thus, our research question is ample, seeking to map what is known about the topic, instead of trying to answer a very specific question (Chang 2018). We followed the recommendations of the PRISMA Extension for Scoping Reviews (Page et al. 2021), which was complemented with the PAGER Framework (Bradbury‐Jones et al. 2022). As we detail below, PAGER is a structured approach for the reporting of findings from scoping reviews based on the identification of Patterns, Advances, Gaps, Evidence for practice and Recommendations for research.

The questions we aimed to address were: In relation to children and young people with SMA:

1. What is known about their everyday experiences and healthcare trajectories and who tells these stories?
2. What kind of narratives regarding their life emerge from the articles and who are the main actors of those narratives?
3. What is the place of the right to health in their stories?

### 2.1 Inclusion Criteria

We included qualitative, quantitative, or mixed methods studies that were published in peer‐reviewed journals between January 2014 and June 2024. The rationale for this timeframe was to have a two‐year ‘pre‐drug’ period of evidence from when the FDA approved the first drug for SMA (Spinraza) in 2016. The studies needed to report the experiences of children and young people (up to 25 years old) with SMA (all types) from their own perspective or that of their parents/family. We included studies published in English and Spanish. If the sample of the studies included other patients with rare diseases, to be included in the review, those with SMA had to represent at least 50% of the sample size.

### 2.2 Exclusion Criteria

We excluded articles that: (i) included participants from the general population or individuals with SMA aged over 25 years; (ii) reported that 50% of their sample comprised adults; (iii) focused on the experiences of pregnant women; and (iv) examined exclusively healthcare professionals' and family caregivers' perspectives.

### 2.3 Search Strategy

We systematically searched four databases to identify relevant studies: (1) Medline (PubMed); (2) Scopus; (3) ProQuest; (4) Scielo (Spanish‐written articles). Search terms were developed in collaboration with the authors and the support of a librarian. Two members of the team created the initial search strategy (MGA‐CQ), based on the PCC Question outline (Population—Concept—Context), which helped identify key terms for the literature search. The strategy was refined by defining each term during the pilot phase (MGA‐CQ‐JC‐MF). This strategy was also adapted to suit the different databases. For example, for PubMed we used the following strategy: “(spinal muscular atrophy OR SMA) AND (children OR childhood OR teenager OR adolescence OR adolescent OR young people OR youth OR families) AND (burden OR narratives OR trajectory OR impact OR perspective) AND (zolgensma OR risdiplam OR spinraza OR onasemnogene abeparvovec‐xioi OR evrysdi OR nusinersen OR drug OR treatment) AND (difficult treatment access OR difficult healthcare access OR health rights OR high cost treatments OR access to healthcare)”. To complement the electronic search, the team reviewed the reference lists of the articles included in the review.

### 2.4 Screening Process

**FIGURE 1:**
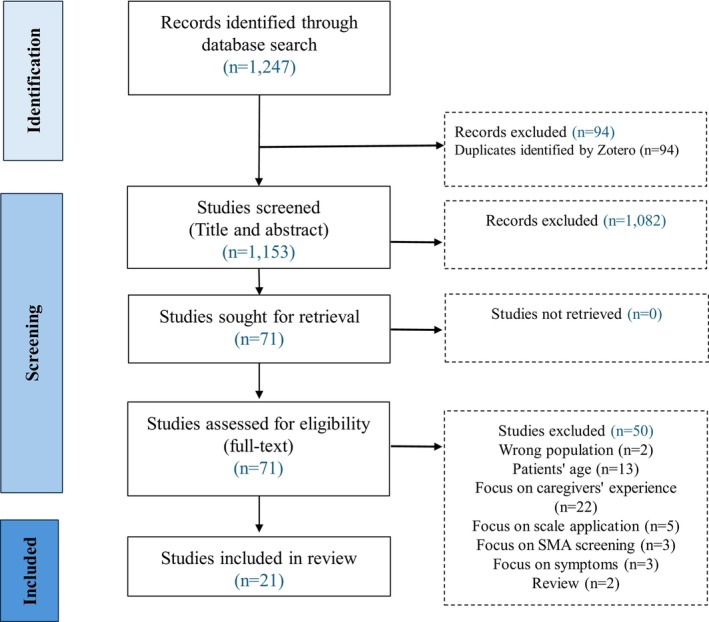
PRISMA flow diagram.

The process comprised two consecutive phases. First, four members of the team screened all the titles and abstracts to remove irrelevant material, eliminating 1082 records out of 1153. The second phase involved the retrieval of 71 articles. After reading the full text and applying the inclusion and exclusion criteria, 50 studies were excluded, and 21 articles were included in the review (see Figure 1).

### 2.5 Data Extraction

Data were extracted by four of the authors (JC‐MF‐MGA‐CQ) using an Excel spreadsheet where each stage of the process was recorded: title and abstract screening, full‐text review, and data extraction. Each of these stages was completed by two reviewers who extracted data independently and then met to resolve doubts and discrepancies. Two senior members of the team reviewed each stage of the process, including data extraction (MGA‐CQ), which focused on identifying the objective, geographical setting, sample, and methodology of each study, as well as the main outcomes.

As this review was concerned with exploring the stories that emerged from the studies, when extracting data, the reviewers used a sociocultural approach to analyse the narratives described in each article, highlighting the social construction of reality (Riessman 2008). This approach proposes that people make sense of their lived experiences through the establishment of past‐present‐future temporal connections when they construct and narrate stories. According to Arthur Frank (1995) and Cheryl Mattingly (1994), the analysis includes two stages. First, the reviewers (JC‐MF‐MGA‐CQ) conducted a plot analysis, where they read each selected article to gain a general understanding of its content and structure. Then, the text was interrogated with some questions: What stories are contained in this article, what are its boundaries, what is the context in which the story takes place, who are the primary and secondary characters, what is the central conflict, and what topics are covered. In the second phase, this analysis was summarised in an excel spreadsheet and it was checked by a second member of the team to solve any discrepancies in the extraction.

### 2.6 The Contribution of the PAGER Framework

After extracting the data, we used the PAGER Framework (Bradbury‐Jones et al. 2021, 2022) to analyse all aspects recorded in our spreadsheet. The first stage involved identifying *Patterns* within the data through an inductive and analytic process. *Patterns* represent the main groupings or emerging themes and show how these are distributed across the included articles, which are summarised in the Patterning Chart (see Table 1). The second stage involves the description of *Advances*, methodological and theoretical, placing the contributions of findings within a historical context. This process enables researchers to consider variations over time and reflect on their implications. The third step focuses on the identification of *Gaps*, which are shaped by the context in which the study is conducted. In our case, the emergence of new high‐cost drugs is an aspect to consider in this discussion. Gaps in knowledge should be specific and address distinct audiences: those who use the literature, such as clinicians or decision‐makers, and those who produce new knowledge (researchers). This is particularly relevant when undertaking reviews in healthcare or educational topics. The fourth stage of the Framework involves mapping *Evidence for practice*. Here, researchers should consider key messages for stakeholders and service providers, as well as implications for their own discipline. Finally, the PAGER Framework offers *Research recommendations*, which consider current Gaps in the literature as well as evidence for practice. Researchers should identify areas of inquiry that have not yet been explored as well as those that do not require further research. The process described above was initially undertaken by the first author with the guidance from two other researchers (CBJ‐JT). This proposal was then reviewed by other members of the team (CQ‐VT). The final version of the analysis is summarised in Table 2.

**TABLE 1:** Patterning chart.

Paper ID	Where is the narrative located?				Tropes about everyday life with SMA			What voices tell the stories?				Right to health discussed		Is the patient an active participant in the study?		Age of person with SMA			SMA type				Sex of parent	
First author	Europe	North America	Western Pacific	LATAM	Odissey	Family journey	Battle	Parents/Carer	Health care provider	SMA	Child/YP with SMA	Yes	No	Yes	No	Under 9	10 to 19 yo	20 and over	> 1 type	I	II	III	Female	Male
Beernaert et al. (2019)	x						x	x	x			x			x				x				50%	50%
Carter et al. (2023)	x				x			x	x	x		x		x		NR	NR	NR	x					
Chen et al. (2021)		x				x		x				x		x				x	x				NR	NR
Fernandes et al. (2022a)				x		x		x	x	x	x		x		x				x				94%	6%
Fernandes et al. (2022b)				x		x		x	x	x	x		x	x			x	x	x				100%	
Flodin (2018)	x					x		x			x		x	x				x			x			
Iyer et al. (2020)		x					x	x	x			x		x				x	x				100%	
Kiefer et al. (2020)	x						x	x		x		x			x					x			100%	38%
Lawton et al. (2015)			x		x			x	x	x			x		x				x				75%	Unclear
Mazzella et al. (2021)		x			x			x		x	x	x		x			x	x	X					
Murrell et al. (2017)		x			x			x	x			x			x					x			95%	58%
Murrell et al. (2018)		x				x		x	x		x	x			x					x			62%	38%
Oude Lansink et al. (2022)	x						x	x	x	x	x		x		x				x				84%	16%
Oude Lansink et al. (2024)	x						x		x		x		x	x			x	x	x					
Pasquini et al. (2021)		x			x			x	x			x			x	x	x	x	x				99%	1%
Pechmann et al. (2022)	x						x	x	x			x			x					x			100%	50%
Qian et al. (2015)		x			x			x		x		x		x		x	x	x	x				77%	23%
Van Kruijsbergen et al. (2021)	x					x		x	x			x			x				x				100%	46%
Wan et al. (2019)			x			x		x		x	x	x		x			x	x	x				33%	67%
Willems et al. (2023)	X					x		x	x			x			x				x				71%	29%
Xiao et al. (2023)		x			x			x		x	x	x			x				x				73%	27%

Abbreviation: NR, not reported.

**TABLE 2:** PAGER framework.

	Patterns	Advances	Gaps	Evidence for practice	Recommendations for research
1	Location of the narrative. There are differences between Global North and Global South in knowledge production	Articles from Latin America (2) are more recent than most others and offer enriching perspectives to global discourse	Scarce evidence about everyday lives and healthcare trajectories from the Global South		Researchers from the Global South are encouraged to explore this topic through the lens of their unique political, historical, and socio‐economic realities
	What voices tell the stories? According to the studies, the most prominently referenced actors are parents or caregivers, with healthcare professionals, the disease, and temporal aspects appearing less frequently	While the primary storytellers are parents and caregivers, articles were identified that seek to elicit patients' voices during the diagnosis process and in some moments of their experience of living with SMA	Individuals with SMA are identified as main actors in only half of the articles. Their voices are included in just 9 out of 21 publications. Notably, children's voices are entirely absent from the literature	Mothers often serve as the primary caregivers for individuals with SMA, making their input essential in the design of services for this population. It is important to recognise and address their own needs as caregivers. Healthcare professionals should, whenever possible, prioritise direct communication with individuals with SMA without relying solely on intermediaries	This review confirms that children and young people are still rarely included as key actors or participants in studies about their own experiences. Future research must address this gap. There is also an urgent need to apply critical lenses of disability and gender in future research
2	The literature reveals three tropes about everyday life with SMA: (1) the odyssey, (2) the family journey and (3) the battle	There is an increasing interest in documenting the lived experiences of young individuals with SMA and their families. Social media has become a key space for connection, information, and collective action, when physical mobility is limited. This opens new directions for exploring digital care networks and community building	Most narratives remain centred in clinical settings, overlooking the everyday life and social dimensions of the illness. Additionally, there is a lack of research exploring the mental health trajectories of children and young people with SMA as they grow up	Healthcare access for patients often unfolds as a complex journey, marked by multiple barriers. Policymakers and healthcare professionals should facilitate smoother care pathways by promoting coordinated, patient‐centred care. Improving communication styles and shared decision‐making processes between professionals and families is also essential	Research needs to explore the everyday lives, values, and identities of young people with SMA, considering their specific historical and geographical contexts. Social sciences and qualitative approaches can contribute to this enquiry. Ethnography or visual methods can aid a deeper understanding of how illness and identity are shaped beyond clinical settings
3	The right to health has become a narrative terrain across articles	Individuals with SMA and their families identify access to information, pharmaceuticals and quality of care as key aspects for their experience when connecting with the health system. Some articles mentioned explicitly concepts such as equality and access to pharmaceuticals with financial protection	Most researchers do not state the right to health explicitly, despite its presence in findings. Parents advocate for the fulfilment of this right or navigate administrative processes to secure it, which implies an extra responsibility when caring for children. This process is carried out with the support of peers or independent research. There is a lack of research to explore the right to health in countries where it becomes contentious	Clinical settings should develop strategies to address this challenge of uncertainty in the relationship that clinicians establish with patients and families, recognising the evolving nature of medical knowledge and showing empathy towards the needs and expectations of patients and their families	To explore the meaning and place of this right in diverse contexts, asking people with SMA what it means for them. To Explore the right to health more explicitly, considering the role of uncertainty in the relationship between clinicians, patients and families. A human rights perspective could help to illuminate future research, as well as an enquiry about the right to live in contexts where pharmaceuticals are not a given

## 3 Results

Following the PAGER Framework, the team explored the literature, searching for Patterns to identify the main findings (see Table 1). These are organised in three categories that inform our research question: (1) Parents as the storytellers of young people's life trajectories; (2) Tropes about everyday life with SMA; (3) The right to health as a narrative terrain. For each category explained below, the research team presents the Advances in the field and Gaps in knowledge, helping to gain a more comprehensive understanding of the topic (see Table 2).

### 3.1 Parents as the Storytellers of Young People's Life Trajectories

#### 3.1.1 Location of New Knowledge

Out of the 21 studies included in this review, 17 were conducted in Northern and Central Europe (*n* = 9) or North America (*n* = 8). Most studies were conducted in the United States (7), followed by Germany (3), Denmark (2), The Netherlands (2), Sweden (1), Ireland (1) and Canada (1). The other four studies reported findings from the Western Pacific region (Australia) and Latin America (Brazil).

These numbers reflect a classic trend of scientific and medical knowledge production originating in the Global North (Montenegro et al. 2020). This brings to the fore an issue of invisibility of everyday experiences from children and youth with SMA from other parts of the globe while reflecting on the socio‐political history of SMA; one which is populated by many “firsts”, including identification of the gene that generates the condition, protocols for newborn screening, gene‐therapy approvals, and technological development.

Moreover, the findings involve a central component of patients' experiences, given by the health system organisation of each of these countries and the means available to access health services and high‐cost pharmaceuticals. Out of the 21 studies, only two were published in a non‐member country of the OECD, Brazil, which applied for membership to this organisation in 2017. It is well‐known that the healthcare system of OECD countries outperformed that of non‐members, ensuring universal health coverage, equitable access and opportunity to healthcare, as well as investing an important percentage of their GDP in health. All these aspects can impact on better healthcare outcomes for their population.

#### 3.1.2 Parents as the Main Story Tellers

The findings of the review show, as a dominant Pattern, that knowledge about the disease and life experiences is mostly constructed from the voices of parents and caregivers, leaving the perspective of the young patients in the background and revealing a significant Gap in the generation of knowledge about their views. In 20 of the 21 articles analysed, parents play a key role in recounting experiences related to diagnosis, treatment, decision‐making, access to information and care, as well as everyday life at home. Studies position parents and caregivers as the means to understand the experience of living with the disease (Mazzella et al. 2021), which could be linked to the very nature of SMA, which in some cases limits the ability of patients to express their experiences independently, especially in the most severe forms of the conditions. As per Advances, our review identified five studies that sought to understand the experience of people with SMA, alongside the accounts of parents and caregivers. Two studies focused on the diagnosis process, examining the implications of early testing (Qian et al. 2015) and diagnosis communication for the lives of young patients and their families (Fernandes et al. 2022b). Other studies explored the plans and personal growth of young people with SMA, beyond the illness (Wan et al. 2019), alongside the physical challenges that SMA implies for social interactions (Oude Lansink et al. 2024), as well as the tension between dependence and the ongoing pursuit of independence (Mazzella et al. 2021).

Despite this advance in knowledge production, the lack of direct representation of patients' voices highlights a limitation in the comprehensive understanding of the disease, resulting in a gap that leaves out a crucial part of the lived experience from the perspective of those living with the disease. Only one article places the patient's experience at the centre of the narrative, following the process of a patient growing up with SMA and her struggle to walk.

In addition to this, another gap highlighted by our review brings to the fore the gender inequalities associated with caregiving. Of the 21 articles reviewed, 18 present the distribution of the sample by sex, and 13 of them identified women as the main caregivers. This information is mainly presented as part of the sampling, without analysing it from a critical perspective, despite the relationship between care work and gender is well established (Duangjina et al. 2025). It is important to point out the Iyer et al. (2020) study, as it highlighted the female presence in the care of people with SMA as a relevant factor due to the ability to expand their carer role from their relative towards other peers on a collective level. The study examined the experience of sharing information about the disease through support groups on social networks, where the figure of the ‘veteran mother’ appears. This term refers to mothers who, with experience in caring for their children with SMA, share information to help other families who are newly diagnosed. According to the author, this action is considered an act of care in itself, as the mother not only cares for her child but also establishes bonds of support and care with peers.

Thus, the results demonstrate that knowledge about the disease comes mainly from the voices of parents and caregivers, with a wide gap with articles that capture the voice of patients singularly. Furthermore, these accounts are mainly positioned in the Global North, revealing a knowledge gap in the Global South. In the Global North, patient experience and disease care are more thoroughly documented, with more developed research addressing everything from diagnosis to the complexities of daily life with SMA, whereas in the Global South, there is an emphasis on the diagnostic process and the initial challenges faced by patients and their families upon receiving an SMA diagnosis.

### 3.2 Tropes About Everyday Life With SMA

The Patterns within stories can be organised around three central tropes, all connected to Frank's typologies, mainly the chaos and the quest narrative. We have named our tropes the odyssey (7 studies), the family journey (8 studies), and the battle (6 studies). These tropes can help us understand what this group values, think, believe, and how individuals respond to their context. While this scoping review is concerned with the experiences of those who have been diagnosed with SMA, their stories emerged in close connection to those of their parents and relatives.

#### 3.2.1 The Odyssey

One of the most prevalent tropes in the reviewed texts is the use of the odyssey analogy to describe the diagnostic process of SMA patients. Seven out of 21 studies described a journey that began with the parents' first suspicions of an illness to the confirmation of the diagnosis. This process is depicted as a long and complex process; in other words, a quest narrative, marked by concerns, anxieties, adversities, lack of knowledge, decision‐making, and a learning process for patients and family members (Carter et al. 2023; Lawton et al. 2015; Mazzella et al. 2021; Murrell et al. 2017; Pasquini et al. 2021; Qian et al. 2015; Xiao et al. 2023). Qian et al. (2015) highlighted that this arduous process affects not only those diagnosed with SMA but also primary caregivers, who take on the role of “navigators” within a healthcare system that does not always facilitate access to appropriate care.

One of the main challenges described by parents is the limited coverage of medical treatments for SMA. Pasquini et al. (2021) explored parents' experiences in seeking the best medical insurance for their children. The authors described this journey as a difficult process in which families must learn to negotiate with insurance companies and navigate the healthcare system. They must understand how insurance works, be aware of required care, and choose the treatment that best suits their situation. This emphasises a highly individualistic healthcare system where access to treatment largely depends on each person's ability to manage their own care (Pasquini et al. 2021). This trope also emerges when patients and families obtain pharmaceutical treatments, describing the process as nonlinear and characterised by mixed emotions—hope, fear, nervousness, sadness, effort, and relief (Xiao et al. 2023).

#### 3.2.2 The Family Journey

This trope is deeply centred on care, highlighting how caregiving becomes the organising principle of family life, as well as the main tool through which families face the many uncertainties brought on by a rare disease. Eight of the reviewed texts focused on analysing the experiences of families and primary caregivers, both in living with the disease and navigating healthcare systems. This is described as crucial to juggle and balance the strengths and weaknesses of healthcare systems. In the context of rare diseases, caregivers are often forced to address gaps in the system while operating in unfamiliar and under‐resourced territories (Chen et al. 2021; Flodin 2018; Murrell et al. 2018; Van Kruijsbergen et al. 2021; Wan et al. 2019; Willems et al. 2023).

One of the most challenging aspects families face in this journey is the way in which they receive and process the diagnosis provided by healthcare professionals. In this regard, Fernandes, Menezes, & Rego (Fernandes et al. 2022a, 2022b) highlighted the importance of communication, specifically the way healthcare professionals deliver this information, as it can have significant consequences on the mental and physical health of patients and their families. Therefore, it is crucial that specialists communicate the diagnosis in a clear fashion, with sensitivity and care. Fernandes et al. (2022a) also emphasised that when families are unprepared for the diagnosis, the likelihood of experiencing post‐traumatic stress increases. In response to systemic shortcomings, families often develop collaborative networks with others living through similar situations. As noted by Murrell et al. (2018), peer networks become vital spaces for sharing knowledge and support, and contribute to enhancing models of family‐centred care.

Moreover, exploring the perspective of parents, caregivers, and patients regarding pharmacological treatments is crucial for understanding their decision‐making process. Two studies explored this topic in relation to Spinraza, analysing how caregivers' perceived needs and concerns about the quality of life of their relatives can impact upon their decision (Chen et al. 2021; Wan et al. 2019).

Based on Frank's typology, this trope reflects some elements of the chaos narrative due to the uncertainty faced by families when caring for their children. It also provides some light on elements related to the quest story, highlighting strategies families use to deal with suffering and lack of knowledge to make decisions.

#### 3.2.3 The Battle

While this trope connects with the quest story in terms of representing the challenges faced by families during the diagnosis and long‐term treatment of SMA, it portrays a specific feeling within individuals, who instead of feeling supported by health systems, find themselves battling against them. The diagnostic process is long, complex, and exhausting, and this notion of ‘battle’ appears in different ways (Iyer et al. 2020; Kiefer et al. 2020; Oude Lansink et al. 2022). One central front is the families' effort to gain access to clear and accurate information about treatment options, crucial for developing care strategies and making informed decisions. Beernaert et al. (2019) stressed the key role of healthcare professionals in conveying this information and involving families in decision‐making. However, Pechmann et al. (2022) showed that, from parents' and caregivers' perspectives, the information provided by healthcare specialists is often insufficient for understanding the disease and coping with it daily. This complicates decisions and impact on the inpatient experience regarding medical treatments (Oude Lansink et al. 2022; Pechmann et al. 2022).

In response, Iyer et al. (2020) have highlighted how families build collective forms of resistance through community encounters and sharing lived experiences. These exchanges provide emotional support, make needs visible, and counter institutional unresponsiveness. Social media emerges as a key space for sharing information and mutual aid, though it also carries risks of misinformation.

The battle also extends to securing pharmaceutical treatments and essential therapies (Kiefer et al. 2020; Oude Lansink et al. 2022). Kiefer et al. (2020) showed how participation in the Expanded Access Program (EAP) in Germany enabled access to life‐saving drugs and provided symbolic hope—allowing families to reimagine the future and construct new perspectives on life with SMA. There is also evidence about how this struggle for treatment access intensified during systemic crises, such as the COVID‐19 pandemic, which exacerbated vulnerabilities. Families were forced to assume greater responsibility for their children's survival, underlining the precariousness of institutional support for rare diseases (Oude Lansink et al. 2022).

Following the PAGER Framework, and based on the narratives presented above, we identify as a Gap the lack of research on children and young people's mental health trajectories as they grow up with SMA. While the focus on parents' well‐being represents an Advance, this area is still developing. Another Gap concerns the predominant emphasis on clinical spaces in young people's narratives, which risks reducing SMA to a purely medical issue. An emerging Advance is attention to social media as a site of connection and information democratisation.

### 3.3 The Right to Health as a Narrative Terrain

While there were no articles included in this review that mentioned the “right to health” in their title or keywords, through the revision of Patterns, we identified 15 papers that discussed one or multiple aspects of it in an explicit or implicit fashion. The review showed that individuals with SMA and their families face challenges in clinical settings due to lack of information (5 studies), coordination of care (6 studies) and barriers in accessing high‐cost drugs (4 studies). Overall, the right to health emerges in connection with the biomedical, geographical and political terrain, as well as with its recent pharmaceutical history.

The need to access clear and detailed information is reported in two main moments of the SMA trajectory, the diagnosis experience (Beernaert et al. 2019; Carter et al. 2023), and when treatment options must be discussed with clinicians (Iyer et al. 2020; Pechmann et al. 2022; Van Kruijsbergen et al. 2021). Access to information involves a relation between patient, relatives, and clinicians, where the training of the latter is mentioned as a barrier or facilitator to ease the patient and family experience. Families reported that the preparedness of clinicians to provide an accurate diagnosis is paramount (Beernaert et al. 2019), as well as their communicational skills to engage with families (Carter et al. 2023; Fernandes et al. 2022b; Van Kruijsbergen et al. 2021). In a Danish study (Beernaert et al. 2019), parents indicated that they were not informed about what SMA entailed (32%), nor about treatment options (18%), nor the fact that their child would have a short life (26%), nor that death was imminent (57%). These findings reflect the relevance of information as a right that can enrich or negatively affect patients' and families' health‐related experiences.

When families did not obtain information from clinicians, they tended to find it by connecting with peers (Pechmann et al. 2022) or through the internet (Iyer et al. 2020). Other patients' experiences were considered highly valuable in understanding the complexity of pharmaceutical treatments. Following the PAGER Framework, a Gap identified from the literature refers to the unexplored role of relatives and patients themselves as advocators to claim the right to health. Moreover, researchers tend to report findings connected to this right without making an explicit statement, which has the potential to obscure said right in the long term.

Regarding accessibility to high‐cost drugs, we identified four studies reporting on the topic in the U.S. (2), Canada (1), and Germany (1). While these are all members of the OECD, a fact that provides insight regarding their level of development and financial resources, access remains problematic. Studies conducted in the U.S. highlight the challenges parents and adult patients face when attempting to obtain insurance coverage for health services and pharmaceutical treatment (Pasquini et al. 2021; Chen et al. 2021). Pasquini argues that access to healthcare has become a convoluted process, as insurance companies have tried to reduce costs at the individual and system level. Additionally, as private health insurance is tied to employment, parents are forced to opt for job opportunities based on health insurance coverage. For relatives and patients engaging with insurance companies, this was a stressful, frustrating, and detrimental experience.

The Canadian study reports an advance in the literature as it uses an explicit equity perspective towards the issue of accessing expensive treatments (Xiao et al. 2023). The authors argue that “consistent and predictable access to disease‐modifying therapies is a major concern for caregivers of children with SMA” (1), a process deeply enmeshed within a regulatory and jurisdictional fabric where funding and eligibility become key concepts. The barriers to accessing drugs impacted deeply on everyday life, and there are reports of families that decided to migrate to Canada to achieve their goal. The unequal access to drugs is also reported in the German study (Kiefer et al. 2020), with parents reporting a sense of injustice for being selected to participate in the special programs while others were denied the opportunity.

A third aspect that echoes the right to health is care coordination, which was discussed in six studies. Overall, they explored the views of patients and families regarding the standards of care they receive and the efforts needed to bridge the gap in healthcare coordination. In a German study (Willems et al. 2023), parents had to become the main articulators of healthcare delivery due to the absence of an institutional coordinator. This was also reported in the U.S., stating that the existence of a healthcare coordinator could ease families' navigation within the system (Murrell et al. 2017, 2018; Qian et al. 2015).

This is particularly relevant for young adults transitioning from paediatric to adult care (Mazzella et al. 2021; Wan et al. 2019), where the lack of coordination (Wan et al. 2019) added stress when losing functionality or experiencing social stigma and self‐esteem issues. The perception of receiving fragmented care influenced young people's decision to withdraw from healthcare services, highlighting a gap between the more person‐centred approach to care delivery in paediatric units and the seemingly impersonal approach in adult settings. Additionally, Mazzella et al. (2021) indicated that young people felt disconnected from healthcare services, as there are no peer‐support groups in the facilities where they seek care. On the other hand, parents indicated that the main barrier to connect with healthcare providers was the lack of empathy regarding their daily experiences, and they reported feelings of loneliness when having to claim their children's rights.

## 4 Discussion

With reference to the PAGER Framework, in this section we discuss the Evidence for practice and Recommendations for research that emerged from the analysis (see Table 2). As these aspects are inextricably linked to the Gaps identified in the previous section, we will reflect on these also.

Overall, the production of knowledge about the experience of SMA is centered in countries of the Global North, which can be explained by several factors, such as the health systems that recognise and provide treatment for the disease, as well as the availability of and access to the drugs. This generates an information‐rich scenario that deserves to be investigated, where different experiences of SMA emerge. Significant advances have been made in understanding the multiple ways of experiencing the disease and its treatment, both in clinical settings and in everyday life. While there is an incipient attempt to record these experiences in the Global South, the biomedical approach dominates the existing narratives, with studies focusing on advances made in clinical settings (Castiglioni et al. 2011; Prado Atlagic et al. 2024). Therefore, a central Recommendation for practice involves the need to explore children and young people's experiences outside the clinic within the Global North. Additionally, there is a need to describe the reality of the Global South, where the resources (structural, human, knowledge, technology and financial) differ from those available in the North, and where the impact of the social determinants of health may also reflect upon this group's experiences. This lack of knowledge has also been highlighted in other Latin American studies (Batista et al. 2024) and in African countries where health inequalities permeate health systems (Tawiah and Sarfo 2025).

Additionally, this research has identified a gap in the understanding of these experiences through an intersectional perspective (Cho et al. 2013), which considers social markers such as gender, age and disability. There is a need to highlight the intersection between (i) patients' gender and that of their caregivers, (ii) the age of research participants and the age groups that are omitted from reports, as well as (iii) the challenges of living with a disability during adolescence and young adulthood. Therefore, future research should focus on attending to this gap through a critical approach. Graells‐Sans et al. (2025) have suggested five dimensions for the incorporation of intersectional theory into nursing practice, with dimensions two and three being central for addressing the Gap mentioned above. First, they suggest that nurses must broaden their disciplinary frameworks to capture a structural perspective. Second, they highlight that understanding inequality as embedded in everyday life is essential to understand individuals' illness experiences in context. Following the same path, and specifically in regard to addressing the needs of children with rare diseases, it has been proposed (Belzer et al. 2022) that an intersectional perspective could allow researchers and clinicians to address the tensions derived from the interactions between home, school, community and healthcare systems.

The findings also present important evidence for practice; on the one hand, mothers emerge as the main caregiver amongst the caregiving figures, which should undoubtedly be considered when designing services to support them. On the other hand, it is important to create space in healthcare guidelines for health professionals to interact directly with their patients, without intermediaries if possible, so that they can express their concerns and wishes directly and privately (Sabetsarvestani and Geçkil 2024). This strategy can positively enhance inpatient experience as the engagement between patients, families and the provider increases (Weng et al. 2024).

From our review, we identified three major narrative tropes: the diagnostic and the odyssey, the family journey, and the battle. While these tropes seem to have a family and local focus, we argue that there is an institutional setting that connects them and generates challenges for individuals and their families. Access to medical treatment, information, and healthcare coverage represents challenges that pose difficulties for learning to navigate the healthcare system in a context marked by little institutional support. As we mentioned earlier, most narratives are set within clinical spaces, obscuring children and young people's experiences outside hospitals. Considering that healthcare access often unfolds as a complex journey, policymakers and healthcare professionals should facilitate smoother care pathways by promoting coordinated, patient‐centred care (Pattison and Corser 2023). Improving communication styles and shared decision‐making processes between professionals and families is essential. Nurses are particularly well suited to implementing these changes due to their teamwork and leadership competencies when working with patients with complex health and social care needs (Karam et al. 2021).

In this sense, a recommendation for research is to develop studies that explore patients' everyday lives, as well as their values, aspirations, and identities, always framed within a specific historical and geographical context (Vindrola‐Padros and Johnson 2014). Listening to and understanding patients' narratives can enable clinicians to establish a deeper engagement with them, enriching both the diagnostic process and treatment (Rushforth et al. 2021) and their overall well‐being.

Another recommendation for research involves highlighting the contribution of social sciences and qualitative approaches as a key for delving deeper into these issues. Through methodologies such as ethnography, visual methods or in‐depth interviews, with a narrative approach that allows one to understand the experiences, beliefs, and actions that shape each patient's journey inside and outside the hospital. Moreover, these approaches allow us to observe how illnesses and patient identities unfold in dynamic and fluid ways (Monaghan and Gabe 2016) contrasting with the notion of illness as a ‘fixed’ identity that constrains how individuals with chronic conditions are represented (Fox and Ward 2008).

While the right to health emerged through the analysis of papers, there were only four articles that discussed it explicitly, using key words such as “inequality” (Xiao et al. 2023), “unmet need” (Chen et al. 2021) or “accessibility” (Mazzella et al. 2021; Pasquini et al. 2021). A scoping review that sought to understand how the principles underlying this right were perceived and used by public health researchers (Montel et al. 2022) reported that while some key principles are well known and used, others remained scarcely explored. The five most assessed principles in the literature include accountability, quality, participation, non‐discrimination, and accessibility, while the less explored comprised access to information, privacy and confidentiality, redress, and informed consent. These findings are relevant for this scoping review as we identified access to information as a key challenge. It has been argued that people need information not only to manage their healthcare but also to improve their health outcomes, becoming a central aspect of people‐centred healthcare (Walsh et al. 2015).

### 4.1 Limitations

We included a small proportion of the literature based on a specific search strategy. While this was robust and systematic, there could be other studies relevant to the topic published in different languages and databases. We privileged the literature that reported studies from the perspectives of people with SMA and their caregivers. However, it is possible that there are relevant insights emerging from papers where healthcare providers reflected on their care of people with SMA and their caregivers. This was beyond the scope of our review but would be an interesting angle for future exploration.

While we tried to track papers published outside of Europe and North America, we found limited literature. Thus, the findings represent a reality focused on the experiences of individuals with SMA and their families living in the Global North. Thus, the findings cannot be used as a standard to represent all SMA cases worldwide. Finally, most papers described the life trajectories of this group within clinical settings, obscuring their experiences outside the clinic. This is an aspect that deserves further enquiry.

## 5 Conclusion

Discussing access to information in the context of rare diseases, and SMA in particular, poses multiple challenges to healthcare providers who navigate clinical settings and build relationships with patients in a context marked by a lack of evidence‐based medical knowledge, scarce clinical protocols and little certainty about the effectiveness and safety of available therapeutic options (Rogalski 2022). This context challenges classical understandings of expert knowledge as both parties learn about the treatment of rare diseases in tandem. Thus, the distinction between patients' knowledge and clinical knowledge becomes blurry in the field, as parents share the illness experience with their children and make most of the crucial decisions regarding their treatment (Henderson et al. 2021; Timmermans and Buchbinder 2015). One of our Recommendations for research involves exploring this topic in a more explicit manner, considering the role of uncertainty in the relationship between clinicians, patients and families. From an Evidence for practice perspective, we believe that clinical settings should develop strategies to deal with this challenge, acknowledging the evolving nature of medical knowledge and showing empathy towards patients' and families' needs and expectations (Fernandes et al. 2022a, 2022b; Flodin 2018; Kiefer et al. 2020; Mazzella et al. 2021; Oude Lansink et al. 2022; Wan et al. 2019; Willems et al. 2023).

According to the findings, the study of the right to health in connection to SMA has mainly been explored in high‐income countries where there are avenues to accessing specialised care for this group. Additionally, the articles had mainly explored the experiences of people with SMA who are insured, either in the public or private healthcare system, leaving the experiences of those lacking coverage unexplored and hidden (Xiao et al. 2023). Considering this gap in knowledge, another recommendation for research involves undertaking studies that consider people from different social, territorial, and ethnic backgrounds from a human rights perspective, as well as expanding the exploration of people's illness experiences to other parts of the world, where the right to health might be placed on more shaky grounds. That is the case of many Latin‐American countries, where there are no systems in place for people to access high‐cost drugs, and they are forced to claim the right to health through legal action (Abadía‐Barrero 2016; Bru 2020).

Finally, we would like to recommend the PAGER Framework as a useful tool to undertake scoping reviews as it helps researchers move from descriptive to analytic approaches when reporting findings. The patterns are particularly useful for looking at data in an approachable manner, allowing researchers to look at particularities without missing the bigger picture.

## Funding

This work was supported by ANID, Fondecyt de Iniciación (Grant ID 11240547).

## Conflicts of Interest

The authors declare no conflicts of interest.

## Data Availability

The data that support the findings of this study are available from the corresponding author upon reasonable request.
